# Supplementary material for: Sketching the SRA: experiment-wide similarity search across 4.8 million metagenomes
Source: bioRxiv. 2026 Oct 7:2026.10.07.757297. Preprint. [Version 1] doi: 10.64898/2026.10.07.757297 (PMC13692301; doi:10.64898/2026.10.07.757297)
Supplement: Supplementary file 1 [file NIHPP2026.10.07.757297v1-supplement-1.pdf]

bioRxiv preprint doi: https://doi.org/10.64898/2026.10.07.757297; this version posted October 8, 2026. The copyright holder for this preprint (which was not certified by peer review) is the author/funder, who has granted bioRxiv a license to display the preprint in perpetuity. It is made available under a CC-BY 4.0 International license.

Supplementary Notes

Sketching the SRA: experiment-wide similarity search across 4.8 million metagenomes

Roland Faure*, Adrita Hossain Nakshi*, Md. Hasin Abrar, Rayan Chikhi, Téo Lemane, Mohsen Taheri, Stephanie Won, Haonan Wu, David Koslicki†, and Paul Medvedev†

bioRxiv preprint doi: https://doi.org/10.64898/2026.10.07.757297; this version posted October 8, 2026. The copyright holder for this preprint (which was not certified by peer review) is the author/funder, who has granted bioRxiv a license to display the preprint in perpetuity. It is made available under a CC-BY 4.0 International license.

Supplementary Note 1: Data summary statistics

Supplementary Table 1: Data summary statistics.

Quantity	Value
Number of metagenome accessions	4,774,811
Size of metagenome sequencing files	1.2 PB (3.1 PBp)
AWS compressed unitig file sizes	723.6 TB (184.1 TB in .zst)
Median number of FMH hashes in each accession	408
Total number of hashes stored in all sketches	193bil
Number of distinct FracMinHash hashes	$\sim$ 33.7bil
Space taken by SourMash representation of all FMH sketches	3.1 TB
Space taken by our representation of all FMH sketches	470 GB
Space taken by all hypervectors	19 GB
Space taken by the full similarity matrix	1.1 TB (842 GB in .zst)
Non-zero entries in the full similarity matrix	697,042,514,619
Space taken by the filtered similarity matrix	90 GB (64 GB in .zst)
Non-zero entries in the filtered similarity matrix	40,521,570,696

bioRxiv preprint doi: https://doi.org/10.64898/2026.10.07.757297; this version posted October 8, 2026. The copyright holder for this preprint (which was not certified by peer review) is the author/funder, who has granted bioRxiv a license to display the preprint in perpetuity. It is made available under a CC-BY 4.0 International license.

Supplementary Note 2: Newly observed 31-mers for soil and human gut metagenomes

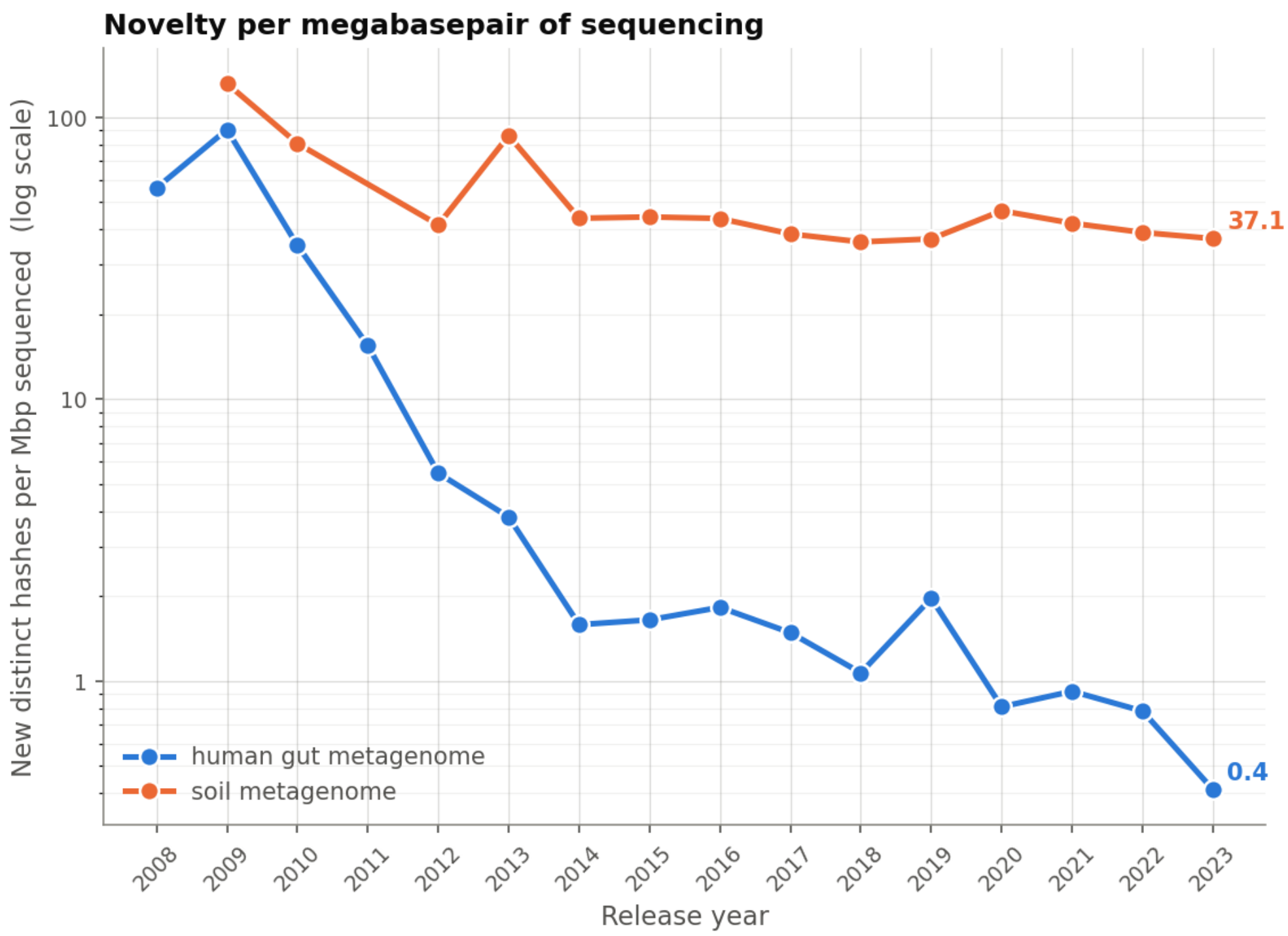

Supplementary Figure 1: **Novel 31-mer hashes returned per megabasepair of sequencing, 2008–2023, for soil and human gut WGS metagenomes.** The log-scale y-axis is the number of such hashes appearing in that environment's WGS runs released that year, divided by the total megabasepairs those runs contributed. Soil is essentially flat across the last decade, from 43.8 novel hashes per Mbp in 2014 to 37.1 in 2023, whereas the human gut rate falls from 1.58 to 0.41 over the same period. By 2023 a megabasepair of soil sequencing returned roughly 90 times more previously-unseen sequence than a megabasepair of human gut sequencing, despite the human gut receiving its largest and deepest sequencing year on record in 2023 (42,098 runs, 188.9 Tbp). Note that the plot does not require a k-mer to appear in multiple years (unlike Figure 2(c,d)), because the focus is on relative, rather than absolute, trajectories.

bioRxiv preprint doi: https://doi.org/10.64898/2026.10.07.757297; this version posted October 8, 2026. The copyright holder for this preprint (which was not certified by peer review) is the author/funder, who has granted bioRxiv a license to display the preprint in perpetuity. It is made available under a CC-BY 4.0 International license.

Supplementary Note 3: Source of error in our Jaccard estimator

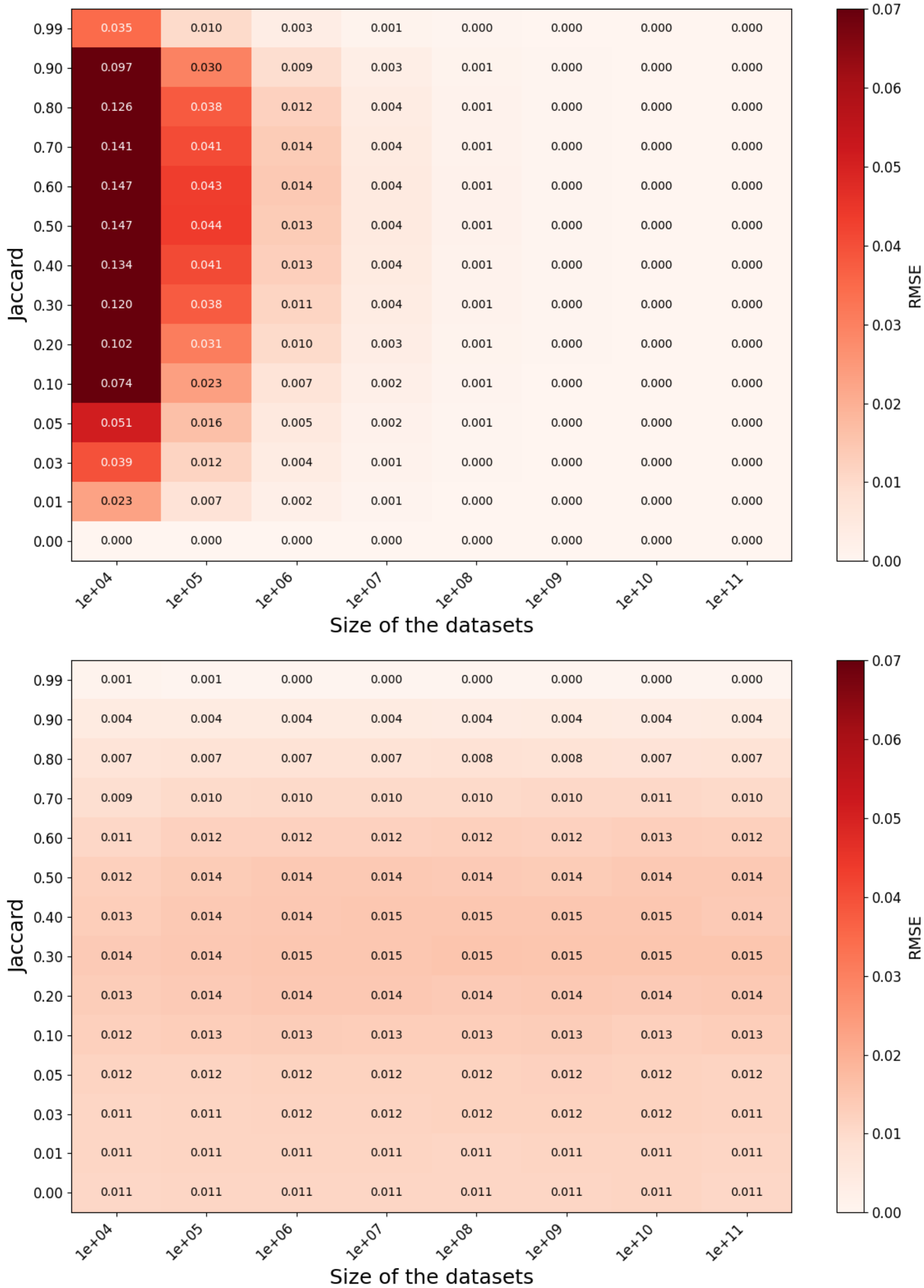

Supplementary Figure 2: **Decomposition of the error of our Jaccard estimator into FracMinHash (top) and Dothash (bottom) errors.** The error is evaluated on pairs of datasets of equal sizes and is reported as the root mean square error (RMSE).

bioRxiv preprint doi: https://doi.org/10.64898/2026.10.07.757297; this version posted October 8, 2026. The copyright holder for this preprint (which was not certified by peer review) is the author/funder, who has granted bioRxiv a license to display the preprint in perpetuity. It is made available under a CC-BY 4.0 International license.

Supplementary Note 4: Metadata correction

Supplementary Table 2: Groups of equivalent `organism` tags, as used in Section 2.3.2. All `organism` tags not mentioned in this table were assigned to a group containing only themselves.

Group Representative	Additional Group Tags
general metagenome	human metagenome, metagenome, Homo sapiens
human blood metagenome	blood metagenome
human gut metagenome	gut metagenome, feces metagenome, human feces metagenome
human lung metagenome	lung metagenome, respiratory tract metagenome, upper respiratory tract metagenome, human tracheal metagenome
human oral metagenome	oral metagenome, human saliva metagenome
human skin metagenome	skin metagenome
human urinary tract metagenome	urine metagenome
human vaginal metagenome	vaginal metagenome
hypersaline metagenome	hypersaline lake metagenome, salt marsh metagenome, salt lake metagenome
sludge metagenome	activated sludge metagenome, bioreactor metagenome, bioreactor sludge metagenome, anaerobic digester metagenome
unidentified	unidentified, uncultured bacterium, uncultured virus, uncultured crAssphage
bovine gut metagenome	bovine metagenome
canine gut metagenome	canine metagenome
fish gut metagenome	fish metagenome
insect gut metagenome	insect metagenome
mouse gut metagenome	mouse metagenome, Mus musculus, Rattus norvegicus
pig gut metagenome	pig metagenome
sheep gut metagenome	sheep metagenome
dust metagenome	indoor metagenome
freshwater metagenome	lake water metagenome, riverine metagenome, drinking water metagenome, groundwater metagenome
hot springs metagenome	hydrothermal vent metagenome
marine metagenome	seawater metagenome, marine plankton metagenome
manure metagenome	poultry litter metagenome
sediment metagenome	freshwater sediment metagenome, marine sediment metagenome
soil metagenome	rhizosphere metagenome, root metagenome, terrestrial metagenome
viral metagenome	human viral metagenome, aquatic viral metagenome, phage metagenome
Equus caballus	Equus asinus asinus
Lassa virus	Mammarenavirus lassaense
Human rhinovirus	Human rhinovirus sp.
SARS-CoV-2	Severe acute respiratory syndrome coronavirus 2
Sinorhizobium	Sinorhizobium meliloti
Treponema pallidum	Treponema pallidum subsp. pallidum SS14, Treponema pallidum subsp. pallidum, Treponema pallidum subsp. pertenue

bioRxiv preprint doi: https://doi.org/10.64898/2026.10.07.757297; this version posted October 8, 2026. The copyright holder for this preprint (which was not certified by peer review) is the author/funder, who has granted bioRxiv a license to display the preprint in perpetuity. It is made available under a CC-BY 4.0 International license.

Supplementary Table 3: Spot-checks of 30 proposed `organism` tag relabelings. The "Type" column refers to the type of relabeling, which is either a refinement (REF) or correction (COR) of the existing label.

Accession	Type	Original Tag	Proposed Tag	Validity	Reason
SRR15051428	REF	metagenome	human gut metagenome	confirmed	
ERR3209818	REF	metagenome	human gut metagenome	rejected	hospital bed rails
ERR2840915	REF	Homo sapiens	human gut metagenome	confirmed	
ERR3322074	REF	Homo sapiens	human gut metagenome	confirmed	
ERR4283234	REF	Homo sapiens	human gut metagenome	confirmed	
ERR10881169	REF	human metagenome	human gut metagenome	confirmed	
ERR2641440	REF	human metagenome	human gut metagenome	confirmed	
ERR6910919	REF	Homo sapiens	human gut metagenome	confirmed	
ERR4596818	REF	metagenome	human oral metagenome	confirmed	
ERR1474606	REF	human metagenome	human oral metagenome	confirmed	
ERR11736193	REF	unidentified	Streptococcus pneumoniae	confirmed	
ERR6161909	REF	general metagenome	wastewater metagenome	rejected	anaerobic digestion reactors
SRR10827708	REF	metagenome	marine metagenome	confirmed	
SRR15526468	REF	metagenome	marine metagenome	confirmed	
ERR5003555	REF	metagenome	marine metagenome	confirmed	
SRR15164790	REF	metagenome	wastewater metagenome	confirmed	
SRR12272182	REF	metagenome	mouse gut metagenome	confirmed	
ERR1414244	REF	metagenome	wastewater metagenome	confirmed	
SRR15744765	REF	metagenome	mouse gut metagenome	confirmed	
ERR10921735	REF	marine metagenome	coral metagenome	confirmed	
ERR3573314	COR	sediment metagenome	wastewater metagenome	confirmed	
ERR4856120	COR	hot springs metagenome	marine metagenome	rejected	highly similar communities in the ocean
SRR17041382	COR	compost metagenome	soil metagenome	rejected	highly similar communities
SRR13618035	COR	air metagenome	pig gut metagenome	rejected	air from swinery
SRR12990121	COR	Mycobacterium leprae	Treponema pallidum	confirmed	
ERR3436327	COR	mouse gut metagenome	human gut metagenome	confirmed	
ERR11510989	COR	mouse gut metagenome	human gut metagenome	rejected	gut transplant human→mouse
ERR10129218	COR	mouse gut metagenome	human gut metagenome	confirmed	
ERR7448812	COR	human gut metagenome	human oral metagenome	confirmed	
ERR3436319	COR	mouse gut metagenome	human gut metagenome	confirmed	

bioRxiv preprint doi: https://doi.org/10.64898/2026.10.07.757297; this version posted October 8, 2026. The copyright holder for this preprint (which was not certified by peer review) is the author/funder, who has granted bioRxiv a license to display the preprint in perpetuity. It is made available under a CC-BY 4.0 International license.

Supplementary Note 5: Filtering the similarity matrix

Supplementary Table 4: Statistics of the similarity matrices under different dataset variations. The first column lists the matrix type: "Full" includes all samples, "WGS only" includes only whole-genome sequencing samples, and "Non-WGS only" includes only non-WGS samples. "$\geq N$ FMH hashes only" denotes matrices constructed from samples containing at least N FracMinHash signatures. "s Jaccard threshold" correspond to matrices including only neighbors with $\geq s$ Jaccard similarity. The last row is the strategy we ultimately chose for our filtered matrix: for an accession X, a neighbor Y with similarity s is retained if $s \geq 0.2$ or fewer than $10,000$ neighbors of X have similarity greater than s.

Type	Size (GB)	N. samples	N. non-zero entries	Mean n. of neighbors	Median n. of neighbors
Full	1,147	4,774,811	697,037,739,808	145,982	58,043
WGS only	26	600,700	17,361,325,130	28,902	2,479
Non-WGS only	1,071	4,174,111	661,685,289,600	158,521	74,329
$\geq 1,000$ FMH hashes only	92	1,252,002	57,480,996,916	45,911	10,446
≥ 500 FMH hashes only	314	2,097,581	198,800,016,376	94,776	33,668
≥ 100 FMH hashes only	988	3,976,546	612,903,068,344	154,130	70,144
≥ 10 FMH hashes only	1,141	4,676,975	695,078,819,348	148,617	62,634
0.2 Jaccard threshold	10	4,774,811	4,000,916,936	838	75
0.1 Jaccard threshold	227	4,774,811	120,142,393,410	25,162	3,809
Top $10,000$ neighbors / Jaccard ≥ 0.2	90	4,774,811	40,516,795,885	8,486	10,518

bioRxiv preprint doi: https://doi.org/10.64898/2026.10.07.757297; this version posted October 8, 2026. The copyright holder for this preprint (which was not certified by peer review) is the author/funder, who has granted bioRxiv a license to display the preprint in perpetuity. It is made available under a CC-BY 4.0 International license.

Supplementary Note 6: Experimental details

6.1 k-mer diversity and novelty

Figure 1 shows how much previously-unseen sequence an environment still returns per unit of sequencing, for the soil and human gut metagenomes. To measure this, we partitioned the corpus by the SRA `organism` metadata field, taken verbatim without harmonization of near-synonyms, and restricted attention to runs with `assay_type=WGS`, and a non-null release date. This yields 165,335 runs (577.7 Tbp) for `human gut metagenome` and 20,723 runs (174.7 Tbp) for `soil metagenome`. A hash is counted as *new* in year Y if Y is the first calendar year in which it appears anywhere in the corpus. We counted these new hashes per environment and divided by the total megabasepairs sequenced across those runs (the SRA `mbases` field, complete for both strata). Note that the resulting rate is not normalised for the fact that the two environments were sequenced at very different scales; this is deliberate, since progressive exhaustion of an environment is precisely a consequence of how heavily it has already been sampled.

6.2 Error analysis

Bias: We evaluated 198 distinct Venn configurations, spanning k-mer set sizes from 10^4 to 10^{10} and Jaccard similarities ranging from 0 to 0.99. For each configuration, we performed 30,000 independent estimations and measured the difference between the true Jaccard and the average value of the estimator.

RMSE measurement: We selected 13 Jaccard values ranging from 0 to 0.99 and 14 dataset sizes ranging from 10^4 to 10^{10}. For each possible configuration of Jaccard and pairs of dataset sizes, we performed 30,000 independent estimations, with which used for downstream error analysis shown in Figure 3(c,d) and Sup. Fig. 2.

Similarity detection false positive rate: To determine the 99.999% prediction interval around Jaccard of zero, we ran 500,000 simulations for each configuration. To run such a large number of simulations, we restricted ourselves to pairs of datasets of the same size.

6.3 Runtime benchmark of Jaccard estimators

Figure 3(b) shows the all-versus-all running times for computing Jaccard similarities across the first 3,536 SRA accessions (sorted alphabetically), using four different tools: `BinDash` v2.5 [39], `sourmash` v2.4.9 [16], `Dashing` v2.1.20 [2], and the hypervector Jaccard estimator we implement. All runs were performed single-threaded on an Intel Core Ultra 9 CPU. For `sourmash`, we used the same parameters as in the rest of the paper (a sampling rate of 1/1000). For *Dashing2* and *BinDash*, we used the default parameters but we set the sketch sizes to a fixed size of 4,096 bytes, matching the hypervector dimensions. We emphasize that this comparison is not intended as a rigorous benchmark, as the methods are not directly comparable: `sourmash` relies on variable-sized FracMinHash sketches, whereas the other tools use fixed-size sketches, and their error distributions differ. Speed vs. accuracy tradeoffs are possible using all methods and we do not explore them in depth. This experiment simply illustrates the computational efficiency of our hypervector-based all-versus-all Jaccard similarity approach and was used as preliminary analysis to determine the choice of software to use.

bioRxiv preprint doi: https://doi.org/10.64898/2026.10.07.757297; this version posted October 8, 2026. The copyright holder for this preprint (which was not certified by peer review) is the author/funder, who has granted bioRxiv a license to display the preprint in perpetuity. It is made available under a CC-BY 4.0 International license.

Supplementary Note 7: Compression of FracMinHash sketch database

The complete FracMinHash sketch collection consisted of 4,774,811 `sourmash` signature files stored in `.sig` format, with a total uncompressed size of 3.1 TB. To make this dataset more practical to store and transfer, we developed a compression pipeline. The compression procedure consists of the following steps.

1. Construction of a global hash table: We first collected all FracMinHash hash values from the input `.sig` files and computed their global union. The union was sorted and deduplicated to produce a single ordered array of unique 64-bit hash values. Let N denote the total number of unique hashes, which in our case was 33.9B. Each hash was assigned a dense identifier from 1 to N based on its position in this sorted array. Therefore, the sorted binary `uint64` array serves as the global hash table, allowing the original hash value to be recovered from its identifier without storing an explicit key-value mapping.

2. Extraction of per-sketch hashes and metadata: For each `sourmash` signature file, we extracted the FracMinHash values from the `mins` field and stored them separately as a binary `uint64` file. The remaining signature information, including sketch parameters and metadata, was saved in a separate metadata directory after clearing the `mins` field, preserving the information needed for later signature reconstruction.

3. Conversion of hash values to dense identifiers: Each per-sketch hash list was then converted to the corresponding list of dense identifiers using the sorted global hash table. Because the global hash table is sorted, this conversion can be performed efficiently by searching for each hash value in the global array. The resulting identifier list represents the same FracMinHash sketch, but uses values from 1 to N rather than the original 64-bit hash values.

4. Compression using Elias-Fano encoding: The per-sketch identifier lists and the global hash table were compressed using Elias-Fano encoding [36]. Elias-Fano is a compression technique that stores a set of u integers from a universe of size U using $\log_2 \frac{U}{u} + 2$ bits per integer. For an accession with n hashes, its per-sketch identifier list takes $n(37 - \log_2 n)$ bits, given that $U = 33.9$B. The global hash table, where $u = 33.9$B and $U = 1.84 \times 10^{16}$, is predicted to take 89 GB. We implemented Elias–Fano encoding using the `bits` succinct data-structure library [12].

5. Final archive compression: Finally, the Elias-Fano compressed per-sketch identifier files, the Elias-Fano compressed global hash table, and the metadata directory were combined and compressed using `xz`.

After compression, the Elias-Fano encoded per-sketch hash identifier files occupied 390 GB, the compressed global hash table occupied 84 GB, and the separated metadata occupied 1.6 GB. The combined size of these components was 475 GB, which was further reduced to 470 GB after `xz` archival compression. Overall, the final compressed database was only 15% of the original `.sig` directory size, corresponding to an approximately 6.7× reduction in storage.

bioRxiv preprint doi: https://doi.org/10.64898/2026.10.07.757297; this version posted October 8, 2026. The copyright holder for this preprint (which was not certified by peer review) is the author/funder, who has granted bioRxiv a license to display the preprint in perpetuity. It is made available under a CC-BY 4.0 International license.

Supplementary Note 8: Hash function implementation details

The FracMinHash (FMH) hashes were generated using the default hash function and seed of Sourmash [16], which is MurmurHash3_x64_128_low64 with a seed of 42. To compute hypergen sketches, recall that we need a function g to map from a 64-bit FMH hashcode to a bitvectors of size $d = 2048$. We implemented g using SplitMix [37, 34], a fast hash function that maps a 64-bit integer to a 64-bit integer that, in practice, mimics full randomness [6]. Let x be a 64-bit FMH hashcode. In our case, $g(x)$ is 2048 bits, or, equivalently, 32 64-bit integers. To obtain $g(x)$, we therefore return the concatenation of 32 SplitMix values, i.e. $\text{SplitMix}(32x) \cdot \text{SplitMix}(32x + 1) \cdots \text{SplitMix}(32x + 31)$. Done in this manner, we guarantee that each any two distinct hashcodes will draw from different random bits. Moreover, our FMH sampling rate of 0.001 guarantees that x is always less than $2^{64} \cdot 0.001$ and hence $32x + 31$ never exceeds 64 bits.

When computing a hypervector of a set of FMH hashcodes (i.e. $h(\mathcal{A})$), we sum the hypervectors of its constituent hashcodes, treating each 0 implicitly as a -1. To store the result, we use a 16-bit integer to store the value of each dimension. We find that 16 bits are sufficient, as the values stored grow with the square root of the number of FMH hashcodes. For example, a dataset containing a billion 31-mers would have a sketch of one million FMH hashcodes, and hence we expect the absolute values of the vectors to be on average 1000, well below the 2^{16} limit.

8.1 Accuracy of Theorem 1

Our confidence intervals rely on Theorem 1 to sample estimator error, which assumes that the underlying hash functions f and g are 4^k-uniform. This is a theoretical ideal of randomness that is not achieved by hash functions in practice. However, since the hash implementations we use are known for having good random properties, we expect our error sampling to be nevertheless accurate. To validate this, we compare our estimator's empirical error distribution with that obtained by the sampling algorithm of Section 4.3.

For each pair of accessions, the error is computed simply as the difference between the true Jaccard (using unitigs) and the estimated Jaccard (using Eq. (1)). However, we are limited in the number of pairs we could sample, as downloading unitigs and computing the Jaccard between them is time consuming. Moreover, we cannot draw pairs uniformly at random, as these are heavily enriched for accessions with Jaccard similarity near 0, whereas we wanted the samples to cover a broad range of s^1, s^2, and s^3.

We therefore take a two stage approach. In the first stage, we create a smaller set of of pairs which is depleted of pairs with zero Jaccard similarity. To do so, we sample pairs uniformly until we can identify and retain 10,000 pairs with $\hat{J} \geq 0.02$. This depleted set creates a truncation bias, i.e. when the true Jaccard lies near 0.02, then only overestimates (e.g., $\hat{J} = 0.03$) are retained, while underestimates (e.g., $\hat{J} = 0.01$) are discarded. Therefore, in the second stage, we compute the ground-truth Jaccard on the depleted set and further exclude pairs with Jaccard less than 0.07. This removes the truncation bias, leaving 724 pairs with computed true Jaccard that we could use for full validation.

For each of the 724 $(\mathcal{A}, \mathcal{B})$ pairs, we start with an already-computed true and estimated Jaccard. Then, we make 10,000 draws from the distribution defined by Theorem 1 to obtain a distribution of $\text{Err}(\mathcal{A}, \mathcal{B})$. We then recorded the decile rank of the error of the estimated Jaccard within this simulated distribution. If the distribution given by Theorem 1 is correct, then these ranks should be uniformly distributed across the ten deciles. For our 724 pairs, the number of pairs in each of the ten deciles was $[57, 68, 70, 79, 61, 73, 83, 85, 74, 74]$. The chi-square test could show no deviation between this empirical distribution and a uniform one (p-value of 0.36). This test confirms that the empirical error of our estimator is consistent with the error computed using simulations in Section 4.3.
